# Fitness Consequences of Advanced Ancestral Age over Three Generations in Humans

**DOI:** 10.1371/journal.pone.0128197

**Published:** 2015-06-01

**Authors:** Adam D. Hayward, Virpi Lummaa, Georgii A. Bazykin

**Affiliations:** 1 Department of Animal and Plant Sciences, Alfred Denny Building, University of Sheffield, Western Bank, Sheffield, S10 2TN, United Kingdom; 2 Institute of Evolutionary Biology, University of Edinburgh, Charlotte Auerbach Road, Edinburgh, EH9 3FL, United Kingdom; 3 Institute for Information Transmission Problems of the Russian Academy of Sciences (Kharkevich Institute), Bolshoy Karetny pereulok 19, Moscow, 127994, Russia; 4 Department of Bioengineering and Bioinformatics, Lomonosov Moscow State University, Vorbyevy Gory 1–73, Moscow, 119992, Russia; 5 Belozersky Institute for Physical and Chemical Biology, Lomonosov Moscow State University, Vorbyevy Gory 1–40, Moscow, 119992, Russia; 6 Pirogov Russian National Research Medical University, Ul. Ostrovityanova 1, Moscow, 117997, Russia; Harbin Medical University, CHINA

## Abstract

A rapid rise in age at parenthood in contemporary societies has increased interest in reports of higher prevalence of *de novo* mutations and health problems in individuals with older fathers, but the fitness consequences of such age effects over several generations remain untested. Here, we use extensive pedigree data on seven pre-industrial Finnish populations to show how the ages of ancestors for up to three generations are associated with fitness traits. Individuals whose fathers, grandfathers and great-grandfathers fathered their lineage on average under age 30 were ~13% more likely to survive to adulthood than those whose ancestors fathered their lineage at over 40 years. In addition, females had a lower probability of marriage if their male ancestors were older. These findings are consistent with an increase of the number of accumulated *de novo* mutations with male age, suggesting that deleterious mutations acquired from recent ancestors may be a substantial burden to fitness in humans. However, possible non-mutational explanations for the observed associations are also discussed.

## Introduction

A growing number of recent studies support the age-related accumulation of *de novo* mutations in the parental germline in humans. Many such mutations are deleterious and have long been speculated to be a substantial contributor to fitness, and a serious public health threat [1,2]. Since counting the number of *de novo* single-nucleotide mutations passed from parents to offspring has become feasible, this number has been shown to be associated with the risk of autism [3–5], schizophrenia [6] and other disorders [7], although the direct measures of mutation load do not always reveal adverse functional correlates, at least in healthy individuals [8]. The majority of new point mutations have a paternal, rather than maternal, origin [1,9,10], in line with a higher rate of germline division in males. Thus, while advanced maternal age is associated with increased risk of chromosomal abnormalities and adverse pregnancy outcomes [11,12], the number of point mutations increases as a function of the age of the father, but is independent of maternal age [10]. Besides single-nucleotide mutations, the number of copy-number variants is also enriched in cases of autism [13] and intellectual disability [14] and increases with paternal age [14], although it is not associated with intelligence in healthy individuals [15].

Despite the evidence for health effects, less is known of the potential fitness consequences of the increase in the number of mutations with paternal age. Paternal age at birth is often associated with child health, and indirect evidence suggests that much of this effect may be due to increased mutation load. For example, increased father’s age is a risk factor for schizophrenia [16,17], autism spectrum disorder [3,16,18–23], and multiple other genetic disorders [24–27], although not for general cognitive ability [28]. Moreover, increased paternal age is associated with reduced Darwinian fitness, largely through its negative association with survival. For instance, increased paternal age was associated with lower life expectancy of daughters in the European royal and noble families [29,30] and in German village genealogies of 15th–early 20th centuries [31]; it was also associated with higher mortality of children in contemporary European cohorts [32–34]. Lastly, a paternal age of over 70 years was associated with lower child survival in the Utah Population Database [35]. However, studies of French [36] and American [37] cohorts of centenarians born in the late 19th century found that they did not have younger fathers than the population average. In addition, a study of a contemporary Canadian population showed that parental age had no effect on frailty and survivorship of the elderly [38], suggesting that most of the paternal age effect is expressed relatively early in life.

While the ancestral age association with offspring fitness is thus proven beyond reasonable doubt, its underlying mechanisms remain unclear. Considering only the ages of the immediate ancestors (father and mother), as in previous studies, is problematic in this respect. First, besides its biological effect, paternal age confounds with unequal trans-generational transmission of resources and wealth within the family that was usually not controlled for in previous studies (i.e., first-borns with on average younger fathers may be favoured or inherit more resources explaining at least part of their increased health or fitness [39]). Second, much of the signal of the possible mutational component of this effect is thus lost. Indeed, unless the acquired mutations are lethal or so deleterious that they prevent reproduction, they can be passed on to the offspring over multiple generations, and many of the deleterious mutations may have little or no phenotypic effect until their number reaches a certain threshold [1]. They can also have incomplete penetrance, low expressivity, or selection against them may be sufficiently weak to allow the individuals carrying them to survive to adulthood and reproduce. Therefore, while each individual may carry a burden of deleterious mutations accumulated over multiple ancestral generations, their effects may be missed when only paternal age is considered as a correlate of ancestral reproductive age. With each ancestral generation, the number of ancestors is doubled, and the mean fraction of the genome contributed by a particular ancestor is halved. All the ancestors combined who are *n* generations away from the proband (the focal individual) are equally likely to pass a new mutation to the proband’s genome, independent of *n*. Therefore, an individual mutation in a proband is as likely to have been acquired from the father as from either of the two grandfathers combined, or from any of the four great-grandfathers. That ancestral ages over multiple generations are important is also highlighted by studies confirming that increased age at childbirth for previous generations of ancestors is associated with increased risk of a number of heritable disorders. For example, increased maternal grandfather’s age was associated with increased risk of schizophrenia [17], and ages of both grandfathers were associated with increased risk of autism, independently of the father’s age [23]. How male ancestral age calculated over several generations is linked with key correlates of fitness such as survival and mating success is however at present unknown, limiting our understanding of how selection against such mutations might operate.

**Fig 1:**
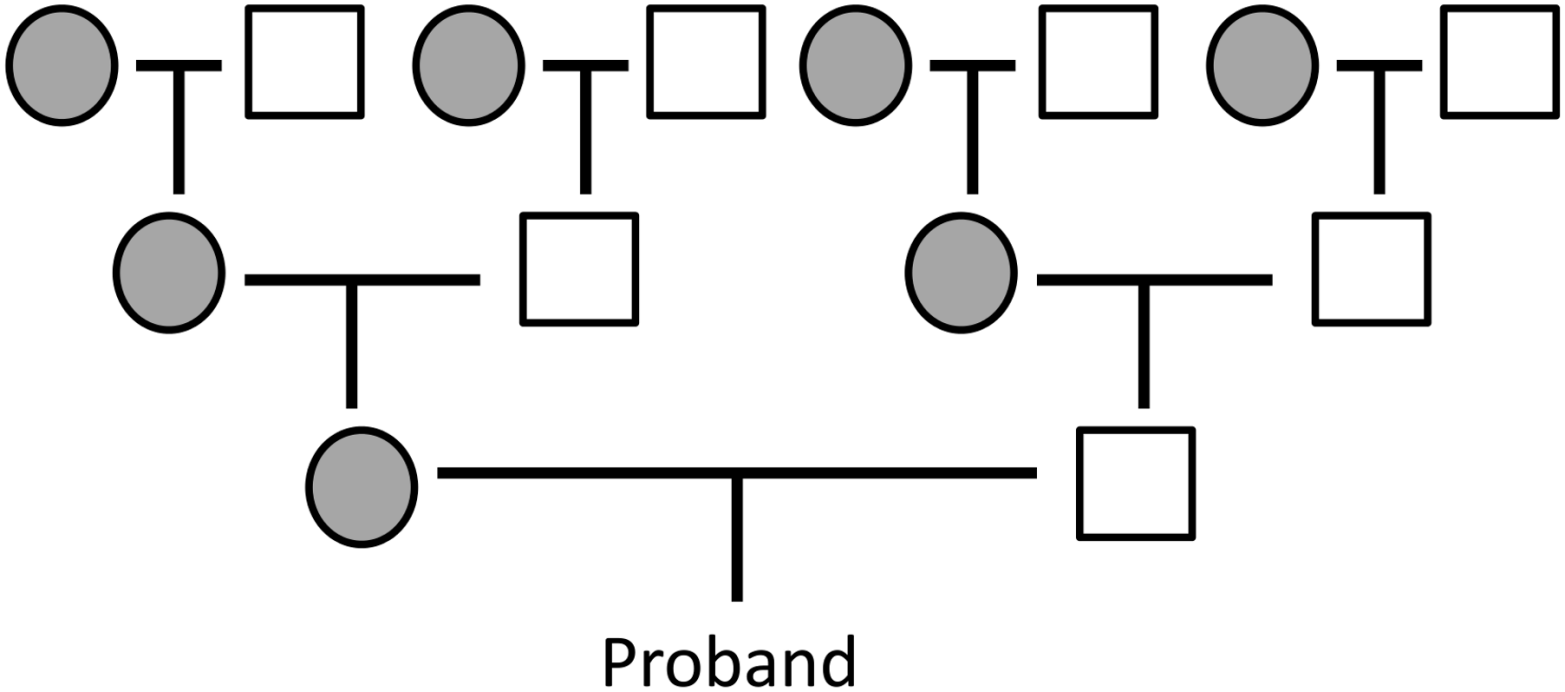
Representation of the data structure for calculation of weighted mean age of male ancestors (WMAMA). The ages at which the male or female proband’s male ancestors (white squares) fathered the proband and the proband’s ancestors were used, weighted by the degree of mean relatedness. Grey circles represent the proband’s female ancestors, whose ages were not used in the calculation (maternal age was controlled for in the models; see Methods). Individuals in our data set had at least both grandfathers known, with varying numbers of great-grandfathers, a factor which was accounted for in the calculation (see Methods).

Here, we use data collected from seven pre-industrial Finnish populations with a detailed pedigree available for several generations [40,41], to investigate whether the combined age of male ancestors over several preceding generations is negatively associated with individual fitness. Longitudinal data from known individuals record their birth, parental identities, socioeconomic status, marriage, reproductive history and death or emigration [42]. This exceptional data set enabled us to estimate the expected change in the number of *de novo* mutations by analysing individuals for whom we know the ages at which they and their ancestors were fathered, whist controlling for confounding factors such as maternal age, social class, birth order (inheritance of wealth), and temporal, spatial and within-family variation in longevity and fecundity (see Methods). For each individual, we measured the weighted mean age of male ancestors (WMAMA) on the basis of the ages at which the proband’s male ancestors and the proband were fathered, up to and including great-grandfather level. In essence, individuals who had older fathers and who had ancestors who themselves had older fathers will have greater WMAMA, with the ages weighted by the relatedness to the proband (Fig 1). We investigated the associations between this estimate of the number of transmitted mutations and several aspects of individual fitness incorporating both survival and reproductive success: (i) survival to the age of 15; (ii) longevity among those that survived to age 15; (iii) whether surviving individuals ever married; (iv) lifetime breeding success in individuals who married. Taken together, the four considered traits thus reflect all aspects of individual postnatal fitness. Child mortality in this population was high, with around 40% of individuals dying before the age of 15, which is similar to contemporary hunter-gatherer populations [43]. Infectious diseases such as smallpox, typhoid, shigellosis and tuberculosis were responsible for a large proportion of deaths, especially in the young [44], and harvest failures and resulting starvation events were common throughout the study period [45]. The population was strictly monogamous, and although paternity cannot be confirmed without genetic data, extra-pair copulation rates are expected to have been low due to strict social regulation of sexual behavior [46], given that extra-pair paternity (EPP) rates based on genetic data are usually <3% in most contemporary European populations [47] with less strict sexual norms. A study using similar historical church record data from Belgium linked to genetic data confirmed EPP rates of <1% [48]. Therefore, estimation of lifetime breeding success using these data is likely accurate also for the male lineage.

## Results

### Survival to age 15

**Fig 2:**
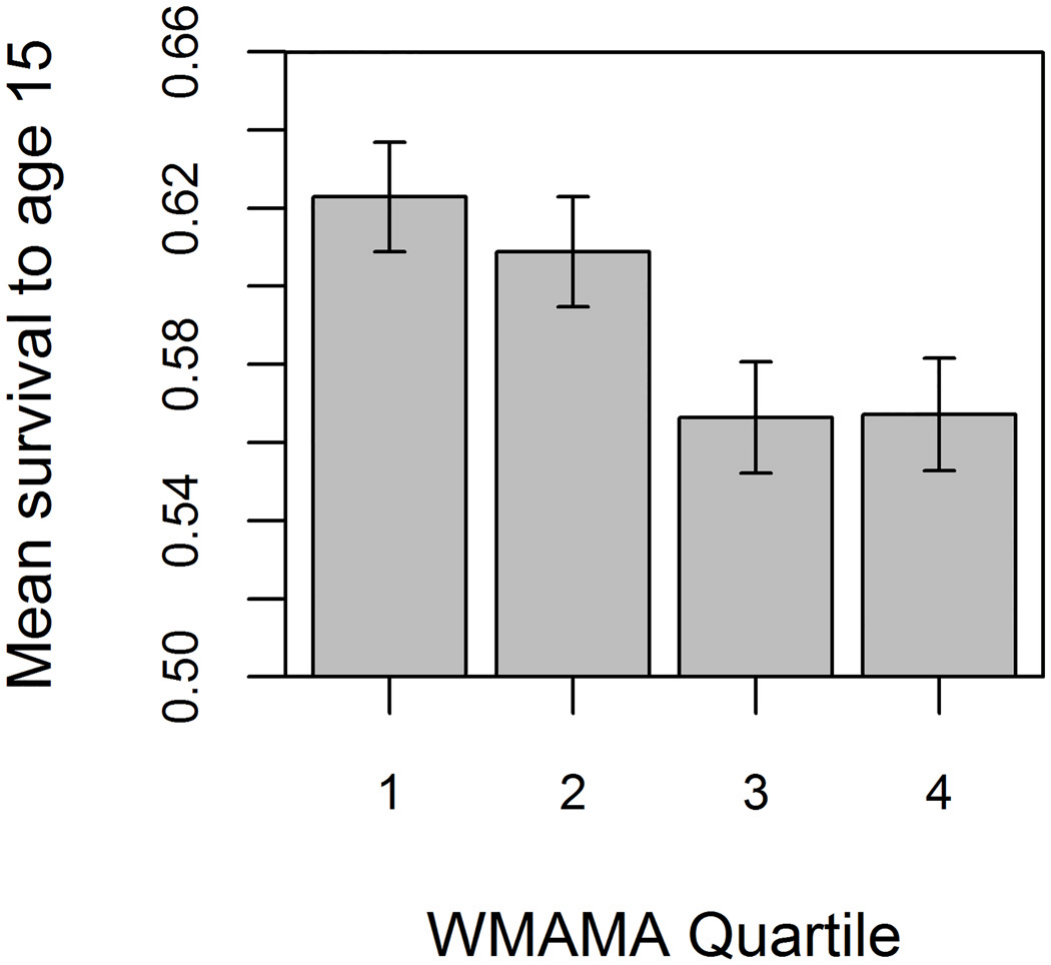
Individuals (n = 4,167) with higher weighted mean age of male ancestors (WMAMA) had lower survival to the age of 15. Bars show mean survival to 15 within each WMAMA quartile, ±1 standard error. The lower threshold of the 2nd, 3rd and 4th WMAMA quartiles are 30.43, 32.92 and 35.86 years respectively.

First, we used generalized linear mixed-effects models (GLMMs) to determine the association between male ancestor age and the probability of survival to age 15 in 4,167 individuals. We found evidence that individuals with older male ancestors over three generations were less likely to survive to adulthood than those with younger ancestors (Fig 2). For instance, individuals whose male ancestors were on average below the age of 30 when they reproduced (20.3% of the sample) had a 62% probability of survival to 15, whereas those whose male ancestors were an average age of 40 or above (6.2% of the sample) had an average survival probability of only 52%. The association remained significant after accounting for other confounding variables: the probability of individuals surviving to age 15 differed between populations, survival was lower in the poor social class than in the rich and middle classes, and twins were less likely to survive to age 15 than singletons (Table 1). The model estimates suggest that, controlling for the other variables, the probability of survival to 15 declines by 13.5% (95% highest probability density interval (HPDI): 0.1%–25.4%) between WMAMA of 30 and 40. Although the estimated posterior distribution has a large variance, the lower boundary of the HPDI is above zero (Table 1), suggesting a statistically robust association.

**Table 1:** The estimated posterior distributions of fixed and random effects from the generalised linear mixed-effects model (GLMM) used to analyse associations between weighted mean age of male ancestors (WMAMA) and survival to age 15.

Variable	Posterior mode	L-95% HPDI	U-95% HPDI
*Fixed effects*			
Intercept	1.6560	1.0051	2.5909
Parish (Hiittinen)	0.0000	0.0000	0.0000
Parish (Ikaalinen)	-0.1428	-0.4391	0.1376
Parish (Jaakkima)	-2.0162	-3.5725	0.1758
Parish (Kustavi)	0.2623	-0.1701	0.6447
Parish (Pulkkila)	-0.6747	-1.0304	-0.3658
Parish (Rautu)	-2.6837	-3.6603	-1.2573
Parish (Tyrvää)	0.3636	0.0274	0.6010
Social (Rich)	0.0000	0.0000	0.0000
Social (Middle)	0.0684	-0.1810	0.2571
Social (Poor)	-0.3261	-0.6516	-0.0156
Twin (0)	0.0000	0.0000	0.0000
Twin (1)	-1.2408	-1.7116	-0.8822
Maternal age	-0.0117	-0.0250	0.0048
WMAMA	-0.0360	-0.0531	-0.0007
*Random effects*			
Birth year	0.1926	0.1338	0.3742
Maternal identity	0.5572	0.3784	0.8433

The posterior modes and Lower and Upper 95% boundaries of the highest probability density intervals (HPDIs) are shown on the logit scale calculated by the model, which analysed data from 4,167 males and females. 95% HPDIs for maternal age overlap zero, but this term was included in the final model since the 95% HPDIs did not overlap zero until WMAMA was included; we therefore wished to account for all possibly important factors associated with survival to 15 when estimating the association with WMAMA. We omitted from this final model any fixed effects which had 95% HPDIs which overlapped zero. See Methods for a full description of fixed effects included in the initial model.

To specifically test whether the effects of WMAMA were limited to certain populations, social classes or either of the sexes, we also fitted models including interactions between WMAMA and proband parish, social class, and sex. We compared these models to that shown in Table 1 using the deviance information criterion (DIC), but by this criterion none of the interaction models were a better fit to the data (S1 Table). This illustrates that the effects of WMAMA were consistent across the populations, social classes and the sexes.

Survival to 15 is a biologically meaningful fitness measure in this population, since 15 is the age of independence and recruitment to the breeding population. However, we also performed a survival analysis, in order to ask a slightly different question: whether WMAMA is associated with variation in mortality risk in a given year of life. We applied a mixed-effects Cox model using the R package ‘coxme’, since a model with a random effect of maternal identity was an improvement on a Cox proportional hazards model with no random effect (χ^2^ = 15.49, df = 1, p < 0.001). The results of the survival analysis (S2 Table) suggested that individuals with older male ancestors had a significantly higher mortality risk, with an increase of 1 year of WMAMA associated with a 1% increase in mortality risk per year of life (compared to model with no WMAMA effect, χ^2^ = 3.92, df = 1, p = 0.048). This was also apparent if father’s age was excluded from the calculation of WMAMA (χ^2^ = 4.15, df = 1, p = 0.042); while father’s age alone, when entered into the model instead of WMAMA, did not significantly influence mortality risk (χ^2^ = 1.12, df = 1, p = 0.291).

### Adult lifespan

Second, we analysed associations between WMAMA and longevity among the 2,465 individuals who were known to survive to at least the age of 15 (GLMM with Poisson errors and a log link function; S3 Table). The association between WMAMA and adult lifespan was negligible and not significantly different from zero (Posterior mean estimate = −0.0010; 95% HPDI = −0.0072–0.0068). No interactions involving WMAMA improved the fit of the model (S4 Table).

### Probability of marriage

**Fig 3:**
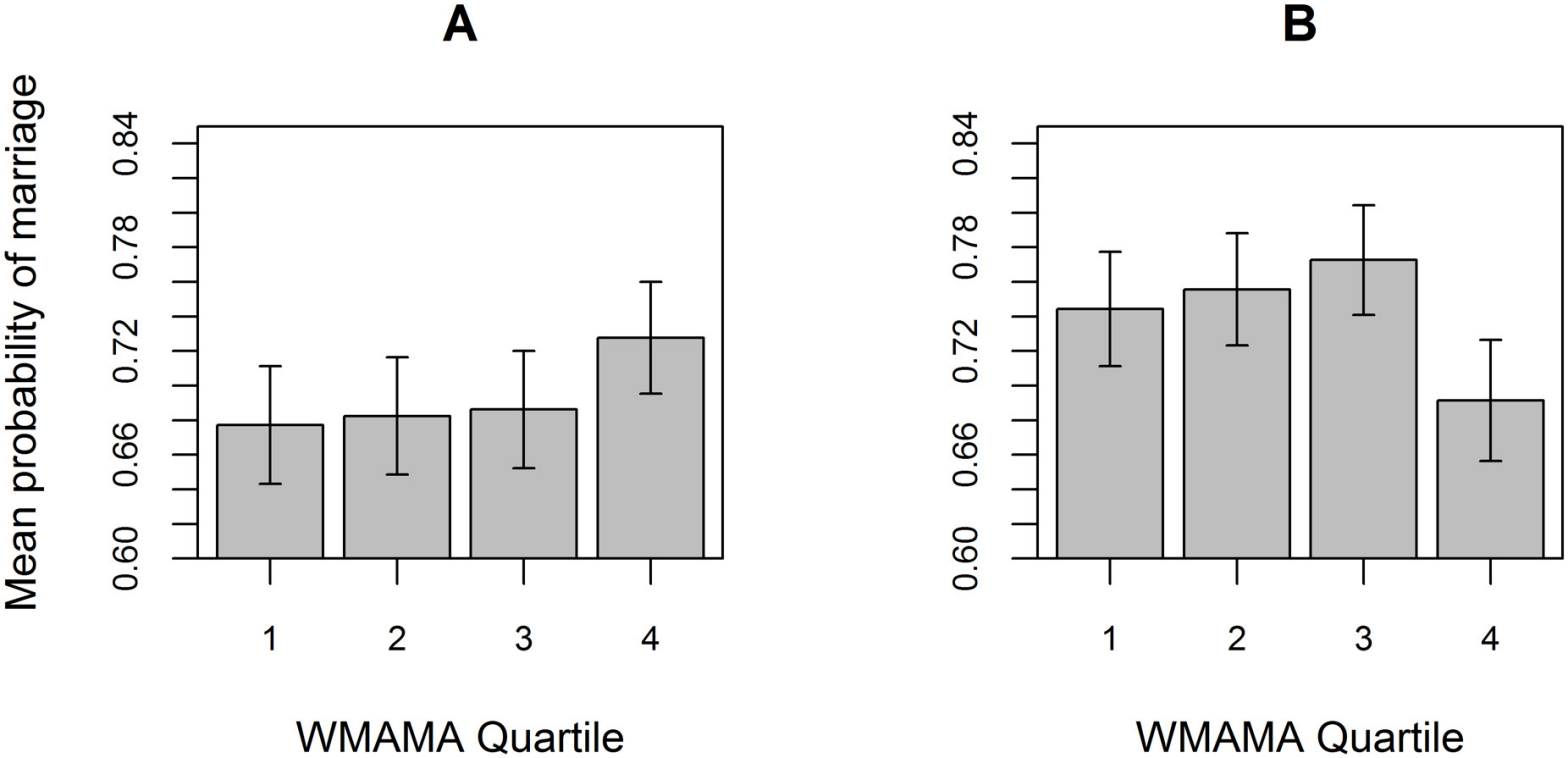
Change in probability of marriage with WMAMA. (A) The probability of a male (n = 757) surviving to the age of 15 marrying was not significantly related to the weighted mean age of male ancestors (WMAMA). However, in females (n = 703), (B) individuals in the highest WMAMA quartile are considerably less likely to marry than those with lower WMAMA. Bars show mean survival to 15 within each WMAMA quartile, ±1 standard error. The lower threshold of the 2nd, 3rd and 4th quartiles are 30.26, 32.81 and 35.80 years in males, and 30.26, 32.92 and 35.66 years in females.

Third, we analysed the association of WMAMA with the probability of marriage among those who survived to the age of 15 and either had a subsequent recorded date of death or, in the case of censored individuals, whose life-history was known to at least until the age of 45 (women) or 50 (men), since these are the ages at which 99% of reproduction has been completed in either sex [49]. We performed separate GLMMs on males (N = 757) and females (N = 703) because of the biological and social differences between the two; males, for instance, marry later than females, and the ability of individuals to secure marriage is linked to different factors in each sex [50]. While the association between WMAMA and probability of marriage was non-significant in males (Fig 3a; S5 Table) (Posterior mode estimate = −0.0120; 95% HPDI = −0.0627–0.0536), a strong and statistically supported negative association was observed in females (Fig 3b; Table 2). The model predicted that, having accounted for significant differences between the parishes in marriage probability, and that individuals from the poor social class were less likely to marry than those from the rich or middle classes (Table 2), the probability of a female marrying drops by 19.6% (95% HPDI: 0.3%–65.9%) between WMAMA of 30 and 40.

**Table 2:** The estimated posterior distributions of fixed and random effects from the generalised linear mixed-effects model (GLMM) used to analyse associations between weighted mean age of male ancestors (WMAMA) and the probability of marriage in females.

Variable	Posterior mode	L-95% HPDI	U-95% HPDI
*Fixed effects*			
Intercept	4.6316	2.1598	7.1415
Parish (Hiittinen)	0.0000	0.0000	0.0000
Parish (Ikaalinen)	0.4561	-0.4388	1.1628
Parish (Kustavi)	-0.1636	-0.9595	0.9853
Parish (Pulkkila)	-108.7254	-133.7155	-20.6428
Parish (Rautu)	-0.4587	-3.5011	3.2916
Parish (Tyrvää)	0.5776	-0.0994	1.3946
Social (Rich)	0.0000	0.0000	0.0000
Social (Middle)	-0.5006	-1.1561	0.0155
Social (Poor)	-1.0906	-2.4251	-0.0832
WMAMA	-0.0965	-0.1562	-0.0156
*Random effects*			
Maternal identity	1.1997	0.0001	2.9799

The posterior modes and 95% boundaries of the highest probability density intervals (HPDIs) are shown on the logit scale calculated by the model, which analysed data from 703 females. We omitted from this final model any fixed effects which had 95% HPDIs which overlapped zero. See Methods for a full description of fixed effects included in the initial model.

### Lifetime breeding success

Finally, we analysed the lifetime breeding success (LBS; total number of children born) in those individuals who were known to marry and to survive to the age of 45 (women) or 50 (men), using a GLMM with Poisson errors and a log link function on 1046 individuals. The posterior estimate of the association with WMAMA, though negative, had 95% HPDI which overlapped zero (S6 Table). There were substantial differences between parishes in lifetime breeding success, and individuals from the poor social class had lower LBS than those from the rich and middle classes, but interactions between WMAMA and parish, social class and sex did not improve the fit of the model (S7 Table).

## Discussion

In this study, we investigated the associations between the age of an individual’s male ancestors and aspects of individual fitness including survival to maturity, ability to secure a mate, longevity, and lifetime breeding success. We aimed to extend previous analyses which have only considered the age of the individual’s father. We found that both males and females with older male ancestors were less likely to survive to 15, and that females with older male ancestors were less likely to marry. Below, we discuss the findings and offer explanations for the observed associations.

We found that an increase in the mean age of paternal ancestors over the three preceding generations was associated with a strong reduction in survival to adulthood (Fig 2). The model predicted that addition of 10 years to the age at which male ancestors gave birth to the lineage of a proband subtracted ~13% from the probability of survival of this proband to the age of 15. The effect was observed independently of the contribution of other factors known to affect early survival, i.e. sex, twin status, social class, maternal age, parish of birth (Table 1). In addition, a high cumulative age of male ancestors was also associated with a reduced likelihood of marriage (assuming survival to reproductive age) among women (Table 2), although not among men. The model predicted that an increase of mean age of male ancestors from 30 to 40 corresponded, for a woman, to a ~20% reduction in the probability of marrying before the age of 45. Again, this effect was independent of the contribution of other factors. We also demonstrated that the effect did not differ significantly between our populations. In all 7 of the analyzed parishes that had associated data on survival to 15, the observed association with WMAMA was negative (S8 Table). Therefore, the data suggests that the results are independently replicable in geographically distinct, but culturally and economically similar, populations.

The data hint that the effect of age of male ancestors on offspring survival may span multiple generations, since there is a negative association between survival and the ages of grandfathers and great-grandfathers. When the ages of the fathers are excluded from calculations of WMAMA, we do not expect the remaining association to be strong. Indeed, the amount of signal is proportional to the variance in the ages of the male ancestors, which goes down by a factor of two each generation (as the number of the considered individuals increases by the factor of two). Therefore, the signal is expected to be in the ratio 1:0.5:0.25 in the fathers, grandfathers and great-grandfathers, respectively. This implies that by removing fathers, we expect to lose between 57% (if the ages of all 6 male ancestors are known) and 66% (if we only know grandfathers’ ages) of the signal. When the age of the proband’s father is excluded from the calculations, the magnitude of the effect of WMAMA on offspring survival drops by a factor of ~2, and although WMAMA is still associated with reduced survivorship, the 95% HPDI around the mode of the posterior estimates overlaps zero (WMAMA excluding father’s age = −0.0166, HPDI = −0.0427–0.0044). However, the same is also true when WMAMA is replaced in the model with father’s age: the age of a proband’s father is negatively associated with their survival to age 15, but the association is weak and the HPDI overlaps zero (posterior mode of father’s age estimates = −0.0087, HPDI = −0.0257–0.0023). The loss of significance is not due to a drop in sample size, since all individuals have fathers of known age. Therefore, grandfather’s and great-grandfather’s ages provide additional information about an individual’s fitness, over and above that of paternal age. The contribution of advanced ages of grandfathers and great-grandfathers to proband survivorship is also underscored by the results of the survival analysis, in which the association with survivorship remains when the father’s age is excluded.

Similarly, we found that WMAMA calculated excluding father’s age was only weakly associated with the probability of a female marrying, and the HPDIs of the estimate overlapped zero (WMAMA excluding father’s age = −0.0326, HPDI = −0.1174–0.0136). In this case, however, replacing WMAMA with father’s age still resulted in an estimate which did not overlap zero (posterior mode of father’s age = -0.0378, HPDI = -0.0680 –-0.0014), suggesting that women with older fathers were less likely to marry, and that the ages of their older male ancestors were less important.

A range of non-mutually exclusive mechanisms could give rise to the observed associations between ancestors’ age and offspring survival, and it is hard to distinguish between them. Socio-economic factors may contribute: for example, fathers that conceive late are more likely to die by the time their offspring reaches adulthood [51], and the same may hold for grandfathers. All our models control for social class of each individual, as well as whether or not they were the first-born in their family and therefore likely to inherit the majority of family wealth. Moreover, although paternal absence is detrimental for child’s well-being in contemporary populations [52], most studies show little or no negative effect of death of fathers or, especially, grandfathers on offspring survival [53]. Indeed, neither father’s [54] nor grandfather’s death [55] are related to increased child mortality risk in the present study population. Still, it is difficult to rule out the possibility that some hard-to-measure or overlooked social or economic variable may stand behind our results. In particular, the observation that father’s age explains the WMAMA effect on female marriage probability suggests that a social, rather than a genetic, link is more plausible for this fitness component. A likely explanation for this is that females born to older fathers could be the last of many children and therefore have several elder sisters. The presence of elder sisters (but not brothers) has been previously linked to decreased marriage probability among females in this population, likely resulting from sibling competition for marriage prospects within the family [50].

Age-related epigenetic changes are also sometimes proposed as the driving force of the parental age effect (e.g. [56]). Epigenetic characteristics of somatic [57–59] as well as germline [60,61] cells change with age; a fraction of these changes could be disruptive, leading to aberrant regulation. Epigenetic changes are associated with some neuropsychiatric disorders [62,63]. Furthermore, some of the epigenetic changes may be transmitted transgenerationally [64]; thus, it has been hypothesized that some of the age-associated epigenetic changes could be passed on to children [65,66] or even grandchildren [66]. Gene imprinting could heritably disable expression of a subset of genes in a parent of origin-dependent way, contributing to the differences between the paternal and maternal effects [67–69]. However, a plausible mechanism by which age-related epigenetic changes may be transmitted transgenerationally is still lacking.

In contrast, the transgenerational transmission of *de novo* mutations is well understood; their accumulation with age leads to a host of genetic disorders, and they likely have considerable consequences for Darwinian fitness. The age effect spanning multiple generations of male ancestors is also consistent with this mechanism. Thus, *de novo* mutations are a likely contributor to the association between ancestral age and offspring fitness shown in this study. However, it is unclear whether *de novo* mutations may explain the observed associations in their entirety. Our analysis suggests that among the parameters that contribute to fitness, survival to adulthood and (for females) probability of marriage are most affected by ancestral age. Survival to reproductive age is the largest component of variation in fitness in most human populations [70], and accounts for the largest share (~35%) of the total opportunity for selection in the studied population [42]. Although we did not find an association between WMAMA and lifetime breeding success, this was estimated only in individuals who had already survived to 15 and who were married; thus, the association with survival to 15 does suggest a large fitness penalty to high male ancestral age. If the effect on fitness is equally divided between the three preceding generations of paternal ancestors, as would be approximately the case for *de novo* mutations, then the estimated ~13% cumulative decrease in survivorship over 3 generations is consistent with a ~4% reduction in fitness per generation in humans when the mean age of male ancestors of a given generation (e.g., father’s age, or mean age of two grandfathers or four great-grandfathers) changes from 30 to 40. The number of new mutations is increased by roughly one-third between father’s ages of 30 and 40 [5,10]. Assuming that their joint effect on fitness is increased similarly (i.e., no epistasis), and that all the observed fitness differences are due to new mutations, a 4% reduction in fitness in progeny of 40-year-old fathers, compared to the 30-year-olds, implies that the per-generation reduction in fitness due to new mutations under relaxed selection, Δ_*R*_, is ~0.12 when males give birth at 30, and ~0.16 when they give birth at 40. The lower and upper boundaries of the 95% HPDI for the reduction in survival would yield Δ_*R*_ of ~0.001 and ~0.25, respectively, when males give birth at 30.

Are such high values of load associated with new mutations consistent with the available data? While *de novo* mutations are gradually becoming an acknowledged factor detrimental to fitness in humans, direct data on their population-level effect on fitness is lacking. Their effect on fitness is also hard to predict through their associations with disorders, because such associations may be very diverse. For example, on the basis of the mutation rates in the father’s germline [10], the prevalence of diseases due to *de novo* loss-of-function single-nucleotide mutations in haploinsufficient genes has been estimated as ~100 to 1,000 per 100,000 births [71]. However, the contribution of recessive or non-coding mutations is unclear, as is how these values translate into population-level differences in fitness.

Δ_*R*_ can be also predicted indirectly as $\Delta_{R}=U\bar{hs}$, where *U* is the rate of deleterious mutations in a diploid genome per generation, and $\bar{hs}$ is the mean selection coefficient against a deleterious heterozygous mutation [72]. Unfortunately, both the fraction of the genome that is functional and the distribution of selection coefficients are known only very approximately. Consider first amino acid-changing mutations. The average *de novo* point mutation rate is 1.2 × 10^-8^ [10], the human genome carries ~3 × 10^7^ coding sites, and approximately 75% of mutations at these sites change the encoded amino acid; therefore, a diploid genome acquires approximately 2 × 1.2 × 10^-8^ × 3 × 10^7^ × 0.75 = 0.54 new amino acid-changing mutations per generation. The distribution of their fitness effects can be estimated from the distribution of allele frequencies at polymorphic sites; however, $\bar{hs}$ is very dependent on the fraction of strongly deleterious mutations, which is hard to estimate because such mutations contribute little to polymorphism [73,74]. For newly arising amino acid-changing mutations, the best-fitting gamma distribution of *hs* yielded $\bar{hs}$ = 0.043 (or less under a different choice of demographic model) [75]. More recent analyses using larger datasets, and accounting for complex demographic history, yielded $\bar{hs}$ = 0.029–0.058 in African Americans, and $\bar{hs}$ = 0.030 in European Americans [73]. These data suggest that the reduction in fitness due to nonsynonymous mutations alone is Δ_*R*P_≈ 0.016–0.031. Predicting the contribution of non-coding mutations is complicated; assuming that 5% of the human genome is under selection [76,77], *U* ≈ 2 × 1.2 × 10^-8^ × 3.2 × 10^9^ × 0.05 = 3.84, with higher estimates for the fraction of genome under selection [78] yielding higher values of *U*. If mutations at functional sites in the entire genome are characterized by the same mean selection coefficients as nonsynonymous mutations at coding sites, these data imply Δ_*R*_ as high as 0.11–0.22. This value is an overestimate if the mean selection against deleterious non-coding mutations is weaker; however, it doesn’t consider other mutation types such as indels, transposable elements insertions and microsatellite instabilities, which may contribute to fitness loss substantially [79].

Finally, Δ_*R*_ can be also obtained, with many caveats, by extrapolating from mutation-accumulation experiments in model species [74]. This yields Δ_*R*_ ≈ 0.0013 (on the basis of experiments in nematodes), or Δ_*R*_ ≈ 0.018 (on the basis of experiments in *Drosophila*), assuming that the mean selection coefficient against deleterious mutations is the same in humans and in these species [74].

Altogether, our estimate of Δ_*R*_ ≈ 0.12 is probably too high to be solely explainable by the load of new mutations. For example, an increase in age from 30 to 40 corresponds to an expected increase of ~20 mutations [10]. If 5% of the genome is functional, then this corresponds to ~1 new deleterious mutation; 4% decrease in fitness over the same period yields the mean $\bar{hs}$ = 0.12 for this mutation, which seems too high. Still, the lower HDPI boundary Δ_*R*_ ≈ 0.001 would be compatible with the available data even if the effect comes exclusively from mutational sources, as it would imply mean $\bar{hs}$ = 0.001.

In summary, we have investigated the associations between the age at reproduction of male ancestors and fitness of focal individuals using a longitudinal data set from a pre-industrial population experiencing natural mortality and fertility. We found that the advanced age of reproduction of ancestors has had a pronounced effect on fitness via reduced likelihood of survival to adulthood, and in addition, for women, a negative association with probability of marriage. These results are a novel demonstration of a transgenerational effect of male age at reproduction on the fitness of their descendants. If the observed effect, or fraction thereof, is mediated by accumulation of *de novo* mutations, it also supports the view (e.g. [80,81]) that the ongoing increase in the mean age of reproduction in modern societies may lead to a higher prevalence of genetic disorders. The detrimental effect of new mutations on fitness may be accumulated over multiple generations. In our ancestors, the loss in fitness occurring each generation due to new deleterious mutations was compensated by purging of deleterious mutations by natural selection. However, if the efficacy of selection in modern human societies is reduced [80,79,82], accumulation of new mutations even over a few generations may have detectable population-level fitness effects. Given the difficulties in distinguishing between alternative explanations for the ancestral age effects, direct assessment of the effect of mutations on fitness through sequencing is necessary to further advance the field.

## Materials and Methods

### Study population and data

We investigated associations between weighted mean age of male ancestors (WMAMA) and several aspects of fitness using longitudinal individual-based data collected from Finnish church records. These data have been used to construct life-histories of individuals from eleven rural populations, referred to as “parishes” [83,84]. We used data collected from seven parishes across Finland: Hiittinen, Ikaalinen, Jaakkima, Kustavi, Pulkkila, Rautu and Tyrvää. The data consist of records of births, marriages and deaths, and for each known individual potential data included: birth date; parental identity; socio-economic status; marriage date; spouse identity; offspring birth date; offspring identity; death date. These data have been used to construct a population pedigree which has been used for quantitative genetic analysis [41], enabling us to record the identity of a focal individual’s ancestors for up to a maximum of ten generations. We only included individuals in the study who were born before 1900 (range: 1688–1899), for whom we knew the father’s age at the birth of the proband and the age of both grandfathers at the birth of the proband’s father and mother. We chose this time interval since this was a period of natural mortality and fertility: this largely agricultural pre-industrial society had poor access to healthcare for all individuals and high child mortality rates across the population. However, there was some important variation in individual wealth and previous work has shown that one of the key determinants of fitness in these populations is social class [85]. Individuals were therefore only included if we knew their social class, which was based on occupation: rich individuals included, for example, farm-owners and merchants; the middle class included occupations such as craftsmen and tenant farmers; the poor class included unskilled individuals such as crofters and labourers [85]. These social classes account for individual differences in wealth, and while we accept that they are not perfect predictors of individual access to resources, they have consistently been associated with variation in many aspects of fitness in many previous studies of these populations. The population was therefore structured based on access to land (landowners, tenant farmers, landless people), but discrepancies between social classes were nevertheless only moderate compared to many other European countries at the time. Marriage between social classes was relatively common, particularly for women [86]. The eldest son usually inherited the parents’ possessions whilst some gifts or dowries were paid for daughters [86].

### Estimation of expected number of mutations

We estimated the number of mutations accumulated by the proband’s lineage over the latest three generations using a formula based on the ages at which the proband’s ancestors and the proband were fathered. For each individual, we recorded the ages at which the proband’s great-grandfathers gave birth to the proband’s grandparents, the grandfathers gave birth to the parents, and the father gave birth to the proband (Fig 1). Since the mean fraction of the genotype acquired from a particular ancestor is halved every generation, the ages of fathers should be given twice as much weight as the ages of grandfathers, and four times the weight of the ages of great-grandfathers. The WMAMA *m* is thus calculated as

$$m=\frac{\sum_{i=1}^{N}a_{i}r_{i}}{\sum_{i=1}^{N}r_{i}},$$ (1)

where *N* is the number of considered ancestors, *a* is the age at birth, and *r* is the coefficient of relationship [87] between the proband and the ancestor: 0.5 for fathers, 0.25 for grandfathers, and 0.125 for great-grandfathers.

### Statistical analysis

We analysed associations between an individual’s estimated WMAMA and four measures of fitness using generalized linear mixed-effects models (GLMMs) in R 3.0.2 using the package MCMCglmm [88]. This powerful iterative Bayesian approach is suitable for our non-Gaussian-distributed data, and provides error estimates by sampling directly from the posterior distribution. For all four fitness measures, we included as fixed effects variables which are known to influence, or which may be associated with, measures of fitness in these populations, including parish as a categorical variable; proband sex; social class as a three-level factor as defined above [85]; twin status as a two-level factor (singleton or twin [89]); birth order as a two-level factor (first-born or subsequent [39]); maternal age as a linear and quadratic covariate [90]. We also included random effects of maternal identity and birth year, to account for variation in survival between families and cohorts respectively. The diagnostics of each model confirmed that effective sample size of each estimate was at least 1000, and that the degree of autocorrelation between consecutive samples for all variables was lower than 0.1. We assessed the suitability of the terms by omitting from the model any fixed effects which had 95% HPDIs which overlapped zero. All remaining terms were retained in all models of WMAMA.

First, we analysed survival to the age of 15 of 4,167 individuals as a binomial trait (0 = died before age 15; 1 = survived) using a GLMM with binomial errors and a logit link function. We arrived at this sample size after applying restrictions to the data as described above. In addition, we excluded individuals who emigrated before the age of 15, or who were born in a different parish before migrating to the focal parish. Social class was assigned on the basis of father’s occupation, since individuals under the age of 15 did not work and so were not assigned their own social class. Models were run for 600,000 iterations, with a burning-in period of 300,000 and a sampling interval of 300 iterations, giving 1000 samples of the posterior distribution.

We also analysed mortality risk across life by performing a survival analysis using the R package ‘coxme’, which allows both fixed and random effects to be included. Initial models included parish, social class, sex and twin status as fixed factors. We also fitted a fixed factor of birth year, divided into 25-year periods (<1700; 1701–1725; …; 1876–1900). We did this because the model would not converge with random effects of both birth year and maternal identity; thus, we fitted birth year as a fixed effect as described and maternal identity as a random effect. We tested these effects by comparing models with and without the fixed effect in question using likelihood ratio tests (LRTs), where the χ^2^ test statistic is calculated as -2*(LogLik_model1_—LogLik_model2_), and the p-value is calculated according to the appropriate number of degrees of freedom. All effects were significant, and so were retained in the model. We then added the main effect of WMAMA, testing it with an LRT. Finally, we re-ran the analyses, replacing WMAMA with father’s age only, and with WMAMA calculated excluding father’s age.

Second, we analysed associations between WMAMA and longevity among the 2,465 individuals who were known to survive to at least the age of 15. Longevity was analysed using a GLMM with Poisson errors and a log link function. The data were restricted as for survival to 15 and also excluded individuals who emigrated before death. Social class was once again assigned on the basis of father’s occupation, so that women who remained unmarried could be assigned a social class. Models were run for 400,000 iterations, with a burning-in period of 200,000 and a sampling interval of 200 iterations, giving 1,000 samples of the posterior distribution.

Third, we analysed the probability of marriage among 757 males and 703 females who survived to the age of 15 using GLMMs with binomial errors and a logit link function (0 = never married; 1 = married). Males and females were analysed separately because of their different life-history schedules and because the cultural and economic factors associated with the probability of marrying differ between the sexes [50]. We arrived at these sample sizes having restricted the data as for analysis of longevity, and once again assigned social class based on father’s occupation. In addition, we did not include individuals who either died or emigrated before the age of 45 and 50 for females and males respectively, since up until these ages individuals are considered reproductive and therefore eligible for marriage. In our population, only 0.5% of individuals who ever married did so for the first time after the age of 50. Models were run for 1 million iterations, with a burning-in period of 400,000 iterations and a sampling interval of 600 iterations.

Finally, we analysed the lifetime breeding success (number of children born) in individuals who were known to marry. We used a GLMM with Poisson errors and a log link function. Data restrictions were applied as for probability of marriage, with the additional restriction that we excluded individuals who died before age 45 (females) or 50 (males) to capture variation in fertility rather than survival. Social class was assigned on the basis of own occupation in the case of males, and on the basis of the first husband’s occupation in females. Models were run for 600,000 iterations, with a burning-in period of 300,000 and a sampling interval of 300 iterations.

In each case, fixed effects were judged to be significantly associated with fitness where the upper or lower 95% highest probability density intervals (HPDIs), the Bayesian equivalent of confidence intervals, of the posterior distribution of the estimates did not overlap zero; those that overlapped zero were described as having no statistical support for an association with fitness. We tested the relative importance of paternal age and the age of grandfathers and great-grandfathers by also (1) fitting models where WMAMA was calculated using only grandfather’s and great-grandfathers ages, and (2) fitting paternal age instead of WMAMA. We also tested for interactions between WMAMA and other fixed effects, assessing the significance of these effects by comparing the DIC (deviance information criterion) of the models with and without the interaction.

## Supporting Information

S1 Table Comparison of generalized linear mixed effects models (GLMMs) investigating associations between weighted mean age of male ancestors (WMAMA) and survival to the age of 15. (DOC)

S2 Table Results of the mixed-effects Cox model of mortality risk in relation to the weighted mean age of male ancestors (WMAMA). (DOC)

S3 Table Posterior estimates for the fixed and random effects on GLMMs of longevity among individuals who survived to at least the age of 15. (DOC)

S4 Table Comparison of GLMMs investigating associations between weighted mean age of male ancestors (WMAMA) and longevity in individuals who survived to age 15. (DOC)

S5 Table Posterior estimates for the fixed and random effects on GLMMs of the probability of marriage for males who survived to at least the age of 15. (DOC)

S6 Table Posterior estimates for the fixed and random effects on GLMMs of lifetime breeding success (LBS) among individuals who survived to at least the age of 15 and who married. (DOC)

S7 Table Comparison of GLMMs investigating associations between weighted mean age of male ancestors (WMAMA) and LBS in individuals who survived to at least the age of 15 and who married. (DOC)

S8 Table Posterior estimates for the fixed and random effects of GLMMs of survival to age 15. (DOC)
