# Supplementary material for: Fitness Consequences of Advanced Ancestral Age over Three Generations in Humans
Source: PLoS One. 2015 Jun 1;10(6):e0128197. doi: 10.1371/journal.pone.0128197 (PMC4451146; doi:10.1371/journal.pone.0128197)
Supplement: S1 Table — (DOC) [file pone.0128197.s001.doc]

**S1 Table. Comparison of generalized linear mixed effects models (GLMMs) investigating associations between weighted mean age of male ancestors (WMAMA) and survival to the age of 15.** The model shown in Table 1, containing the fixed effect of WMAMA, is not improved by the interactions with parish, social class or sex, as seen from the deviance information criteria (DIC) of the four models.

| **Model** | **DIC** | **ΔDIC** |
| --- | --- | --- |
| ***WMAMA*** | ***5315.47*** | ***0.00*** |
| WMAMA x Parish | 5316.21 | 0.74 |
| WMAMA x Social | 5317.24 | 1.03 |
| WMAMA x Sex | 5319.01 | 1.77 |
