# Supplementary material for: Fitness Consequences of Advanced Ancestral Age over Three Generations in Humans
Source: PLoS One. 2015 Jun 1;10(6):e0128197. doi: 10.1371/journal.pone.0128197 (PMC4451146; doi:10.1371/journal.pone.0128197)
Supplement: S2 Table — (DOC) [file pone.0128197.s002.doc]

**S2 Table. Results of the mixed-effects Cox model of mortality risk in relation to the weighted mean age of male ancestors (WMAMA).** Hazard refers to the mortality risk of the group relative to the reference group, with the associated standard error. χ² statistics refer to the result of a likelihood ratio test (LRT) comparing the full model with a model with the term in question dropped, with the associated p-value. Degrees of freedom applied to the LRT are shown in subscript parentheses.

| **Variable** | **Hazard** | **SE** | **χ²** | **p** |
| --- | --- | --- | --- | --- |
| *Fixed effects* |  |  |  |  |
| Parish (Hiittinen) | 0.0000 | 0.0000 | 45.62_(6)_ | <0.001 |
| Parish (Ikaalinen) | 1.0177 | 0.0641 |  |  |
| Parish (Jaakkima) | 2.3364 | 0.3490 |  |  |
| Parish (Kustavi) | 0.9877 | 0.0888 |  |  |
| Parish (Pulkkila) | 1.1394 | 0.0781 |  |  |
| Parish (Rautu) | 3.8659 | 0.2156 |  |  |
| Parish (Tyrvää) | 0.8986 | 0.0641 |  |  |
| Social (Rich) | 0.0000 | 0.0000 | 4.62_(2)_ | 0.099 |
| Social (Middle) | 0.9127 | 0.0480 |  |  |
| Social (Poor) | 1.0336 | 0.0751 |  |  |
| Sex (Male) | 0.0000 | 0.0000 | 12.14_(1)_ | <0.001 |
| Sex (Female) | 0.8757 | 0.0381 |  |  |
| Twin (0) | 0.0000 | 0.0000 | 32.63_(1)_ | <0.001 |
| Twin (1) | 1.7750 | 0.0942 |  |  |
| Birth year (<1700) | 0.0000 | 0.0000 | 78.02_(8)_ | <0.001 |
| Birth year (<1725) | 0.8722 | 0.8205 |  |  |
| Birth year (<1750) | 1.4755 | 0.7613 |  |  |
| Birth year (<1775) | 1.5954 | 0.7439 |  |  |
| Birth year (<1800) | 1.8567 | 0.7393 |  |  |
| Birth year (<1825) | 1.7534 | 0.7377 |  |  |
| Birth year (<1850) | 1.6457 | 0.7374 |  |  |
| Birth year (<1875) | 1.5934 | 0.7372 |  |  |
| Birth year (<1900) | 1.0477 | 0.7380 |  |  |
| WMAMA | 1.0100 | 0.0051 | 3.92_(1)_ | 0.048 |
| *Random effects* |  |  |  |  |
|  | Std. Dev. | Variance |  |  |
| Maternal identity | 0.2627 | 0.0690 |  |  |
