# Supplementary material for: Fitness Consequences of Advanced Ancestral Age over Three Generations in Humans
Source: PLoS One. 2015 Jun 1;10(6):e0128197. doi: 10.1371/journal.pone.0128197 (PMC4451146; doi:10.1371/journal.pone.0128197)
Supplement: S3 Table — (DOC) [file pone.0128197.s003.doc]

**S3 Table.** **Posterior estimates for the fixed and random effects on GLMMs of longevity among individuals who survived to at least the age of 15.** Estimates are shown as derived from the Poisson model including weighted mean age of male ancestor (WMAMA). The model was used to analyse the longevity of 2,996 individuals.

| **Variable** | **Posterior mode** | **L-95% HPDI** | **U-95% HPDI** |
| --- | --- | --- | --- |
| *Fixed effects* |  |  |  |
| Intercept | 3.8307 | 3.5884 | 4.0632 |
| Parish (Hiittinen) | 0.0000 | 0.0000 | 0.0000 |
| Parish (Ikaalinen) | -0.0285 | -0.1285 | 0.0519 |
| Parish (Jaakkima) | -0.2430 | -0.7563 | 0.2834 |
| Parish (Kustavi) | 0.0776 | -0.0338 | 0.1779 |
| Parish (Pulkkila) | -0.3205 | -0.4546 | -0.1900 |
| Parish (Rautu) | -0.3488 | -0.7506 | 0.0615 |
| Parish (Tyrvää) | 0.0818 | 0.0184 | 0.1723 |
| Sex (Male) | 0.0000 | 0.0000 | 0.0000 |
| Sex (Female) | 0.0536 | -0.0007 | 0.0995 |
| Twin (0) | 0.0000 | 0.0000 | 0.0000 |
| Twin (1) | 0.1875 | 0.0198 | 0.3598 |
| WMAMA | -0.0010 | -0.0072 | 0.0068 |
| *Random effects* |  |  |  |
| Birth year | 0.0007 | 0.0002 | 0.0052 |
| Maternal identity | 0.0413 | 0.0293 | 0.0638 |
| Residual | 0.1534 | 0.1338 | 0.1708 |
