# Supplementary material for: Fitness Consequences of Advanced Ancestral Age over Three Generations in Humans
Source: PLoS One. 2015 Jun 1;10(6):e0128197. doi: 10.1371/journal.pone.0128197 (PMC4451146; doi:10.1371/journal.pone.0128197)
Supplement: S4 Table — (DOC) [file pone.0128197.s004.doc]

**S4 Table.** **Comparison of GLMMs investigating associations between weighted mean age of male ancestors (WMAMA) and longevity in individuals who survived to age 15.** The model shown in S3 Table, containing the fixed effect of WMAMA, is not improved by any of the interactions, as seen from the deviance information criteria (DIC) of the models.

| **Model** | **DIC** | **ΔDIC** |
| --- | --- | --- |
| ***WMAMA*** | ***9992.84*** | ***0*** |
| WMAMA x Parish | 9993.36 | 0.52 |
| WMAMA x Sex | 9994.24 | 0.88 |
