# Supplementary material for: Fitness Consequences of Advanced Ancestral Age over Three Generations in Humans
Source: PLoS One. 2015 Jun 1;10(6):e0128197. doi: 10.1371/journal.pone.0128197 (PMC4451146; doi:10.1371/journal.pone.0128197)
Supplement: S5 Table — (DOC) [file pone.0128197.s005.doc]

**S5 Table.** **Posterior estimates for the fixed and random effects on GLMMs of the probability of marriage for males who survived to at least the age of 15.** Estimates are shown as derived from a model including weighted mean age of male ancestors (WMAMA) with binomial errors and a logit link function. The model was used to analyse the probability of marriage of 757 males.

| **Variable** | **Posterior mode** | **L-95% HPDI** | **U-95% HPDI** |
| --- | --- | --- | --- |
| *Fixed effects* |  |  |  |
| Intercept | 1.7576 | -0.0875 | 3.9060 |
| Parish (Hiittinen) | 0.0000 | 0.0000 | 0.0000 |
| Parish (Ikaalinen) | -0.2684 | -0.8837 | 0.5209 |
| Parish (Kustavi) | -0.2685 | -1.0285 | 0.5808 |
| Parish (Pulkkila) | -246.7763 | -307.0588 | -188.8352 |
| Parish (Rautu) | 0.4255 | -5.4580 | 3.6184 |
| Parish (Tyrvää) | 1.0031 | 0.2634 | 1.5787 |
| Birth order (1) | 0.0000 | 0.0000 | 0.0000 |
| Birth order (2) | -0.7823 | -1.5109 | -0.2253 |
| WMAMA | -0.0120 | -0.0627 | 0.0526 |
| *Random effects* |  |  |  |
| Maternal identity | 0.8936 | 0.0001 | 2.8107 |
