# Supplementary material for: Fitness Consequences of Advanced Ancestral Age over Three Generations in Humans
Source: PLoS One. 2015 Jun 1;10(6):e0128197. doi: 10.1371/journal.pone.0128197 (PMC4451146; doi:10.1371/journal.pone.0128197)
Supplement: S6 Table — (DOC) [file pone.0128197.s006.doc]

**S6 Table. Posterior estimates for the fixed and random effects on GLMMs of lifetime breeding success (LBS) among individuals who survived to at least the age of 15 and who married.** Estimates are shown as derived from the Poisson model including weighted mean age of male ancestor (WMAMA). The model was used to analyse the LBS of 1,046 males and females.

| **Variable** | **Posterior mode** | **L-95% HPDI** | **U-95% HPDI** |
| --- | --- | --- | --- |
| *Fixed effects* |  |  |  |
| Intercept | 1.4282 | 1.0296 | 1.7653 |
| Parish (Hiittinen) | 0.0000 | 0.0000 | 0.0000 |
| Parish (Ikaalinen) | 0.3155 | 0.1779 | 0.4231 |
| Parish (Kustavi) | -0.1109 | -0.2909 | 0.0512 |
| Parish (Rautu) | -0.3843 | -1.1552 | 0.3168 |
| Parish (Tyrvää) | 0.2043 | 0.1099 | 0.3288 |
| Social (Rich) | 0.0000 | 0.0000 | 0.0000 |
| Social (Middle) | -0.0480 | -0.1330 | 0.0562 |
| Social (Poor) | -0.2656 | -0.4337 | -0.0720 |
| WMAMA | -0.0029 | -0.0146 | 0.0067 |
| *Random effects* |  |  |  |
| Birth year | 0.0010 | 0.0002 | 0.0276 |
| Residual | 0.2641 | 0.2136 | 0.3149 |
