# Supplementary material for: Fitness Consequences of Advanced Ancestral Age over Three Generations in Humans
Source: PLoS One. 2015 Jun 1;10(6):e0128197. doi: 10.1371/journal.pone.0128197 (PMC4451146; doi:10.1371/journal.pone.0128197)
Supplement: S7 Table — (DOC) [file pone.0128197.s007.doc]

**S7 Table.** **Comparison of GLMMs investigating associations between weighted mean age of male ancestors (WMAMA) and LBS in individuals who survived to at least the age of 15 and who married.** The model shown in S6 Table, containing the fixed effect of WMAMA, is not improved by any of the interactions, as seen from the deviance information criteria (DIC) of the models.

| **Model** | **DIC** | **ΔDIC** |
| --- | --- | --- |
| WMAMA | 5078.77 | 0.00 |
| WMAMA x Parish | 5077.01 | -1.76 |
| WMAMA x Social | 5078.25 | -0.52 |
