# Supplementary material for: Fitness Consequences of Advanced Ancestral Age over Three Generations in Humans
Source: PLoS One. 2015 Jun 1;10(6):e0128197. doi: 10.1371/journal.pone.0128197 (PMC4451146; doi:10.1371/journal.pone.0128197)
Supplement: S8 Table — (DOC) [file pone.0128197.s008.doc]

**S8 Table. Posterior estimates for the fixed and random effects of GLMMs of survival to age 15.** The model contains an interaction between weighted mean age of male ancestors (WMAMA) and Parish which did not improve the fit of the model as assessed by its DIC value compared to the model without the interaction (S1 Table). Estimates are shown as derived from the Poisson model.

| **Variable** | **Posterior mode** | **L-95% HPDI** | **U-95% HPDI** |
| --- | --- | --- | --- |
| *Random effects* |  |  |  |
| Intercept | 0.7479 | -0.6921 | 2.6624 |
| Parish (Hiittinen) | 0.0000 | 0.0000 | 0.0000 |
| Parish (Ikaalinen) | 0.3013 | -2.1017 | 2.1234 |
| Parish (Jaakkima) | 34.7281 | -0.2587 | 85.8880 |
| Parish (Kustavi) | 1.5665 | -1.6178 | 4.4455 |
| Parish (Pulkkila) | 1.3312 | -1.3948 | 3.8255 |
| Parish (Rautu) | -2.5263 | -11.8650 | 8.0843 |
| Parish (Tyrvää) | 1.6379 | -0.3002 | 4.1884 |
| Social (Rich) | 0.0000 | 0.0000 | 0.0000 |
| Social (Middle) | 0.0196 | -0.1647 | 0.2484 |
| Social (Poor) | -0.3302 | -0.6714 | 0.0128 |
| Twin (0) | 0.0000 | 0.0000 | 0.0000 |
| Twin (1) | -1.3034 | -1.6824 | -0.7716 |
| Maternal age | -0.0057 | -0.0236 | 0.0071 |
| WMAMA | 0.0080 | -0.0553 | 0.0442 |
| Parish (Hiittinen):WMAMA | 0.0000 | 0.0000 | 0.0000 |
| Parish (Ikaalinen):WMAMA | -0.0030 | -0.0621 | 0.0624 |
| Parish (Jaakkima):WMAMA | -0.9823 | -2.5171 | -0.0671 |
| Parish (Kustavi):WMAMA | -0.0485 | -0.1243 | 0.0562 |
| Parish (Pulkkila):WMAMA | -0.0598 | -0.1293 | 0.0267 |
| Parish (Rautu):WMAMA | -0.0797 | -0.2968 | 0.2818 |
| Parish (Tyrvää):WMAMA | -0.0418 | -0.1163 | 0.0164 |
| *Random effects* |  |  |  |
| Birth year | 0.2044 | 0.1128 | 0.3496 |
| Maternal identity | 0.5375 | 0.3575 | 0.8378 |
